# Steady Shear Rheology of Suspensions of Mixtures of Starch Nanoparticles and Cellulose Nanocrystals

**DOI:** 10.3390/nano15130966

**Published:** 2025-06-22

**Authors:** Hanie Alizadeh, Rajinder Pal

**Affiliations:** Department of Chemical Engineering, University of Waterloo, Waterloo, ON N2L 3G1, Canada; halizadeh@uwaterloo.ca

**Keywords:** rheology, viscosity, suspensions, cellulose nanocrystals, nanocrystalline cellulose, starch nanoparticles, nanoparticles, non-Newtonian, nanofluid, power-law model

## Abstract

The steady shear rheology of suspensions of mixtures of rod-shaped cellulose nanocrystals (NCC) and spherical starch nanoparticles (SNPs) was investigated experimentally over a broad range of NCC and SNP concentrations. The NCC concentration varied from about 1 to 6.7 wt% and the SNP concentration varied from 5 to 30 wt%. The suspensions of mixtures of NCC and SNPs were pseudoplastic (shear-thinning) in nature. The viscous behavior of suspensions of mixtures of NCC and SNPs could be described adequately using the power-law model. The power-law parameters, that is, consistency index and flow behavior index, were dependent on the concentrations of both NCC and SNPs. The consistency index increased substantially with increases in NCC and SNP concentrations. The flow behavior index generally decreased with an increase in NCC and SNP concentrations; that is, the suspension mixtures became more shear-thinning with increases in NCC and SNP concentrations. However, the dependence of the consistency index and flow behavior index on NCC concentration was much stronger as compared with the SNP concentration.

## 1. Introduction

Thickening agents (thickeners or rheology modifiers) are materials which, when added to a liquid, increase its viscosity and modify its rheology. The thickening and/or modification of the rheology of liquids is required in many practical applications dealing with pharmaceuticals, foods, cosmetics, drilling fluids, and many more [1,2,3,4]. The worldwide market for thickening agents is more than USD 10 billion on an annual basis [4]. As an example, thickening of liquids is used as a medical dietary adjustment for individuals with dysphagia (difficulty in swallowing) to prevent choking and keep liquid food from entering the airways [5]. In drilling fluids, thickeners are used as rheological performance regulators. Thickeners are also used extensively in the formulation of suspensions and emulsions [1]. Suspensions are dispersions of solid particles in liquids whereas emulsions are dispersions of liquid droplets in another immiscible liquid. The dispersed particles/droplets of suspensions and emulsions are prone to phase separation under the influence of gravity. The heavier particles or droplets settle at the bottom of the vessel whereas lighter particles and droplets cream at the top of the container. To minimize sedimentation and creaming in suspensions and emulsions, thickeners are added to the continuous phase of the dispersion to increase the continuous-phase viscosity. The settling or upward rise velocity of particles/droplets is inversely related to the viscosity of the continuous-phase liquid, as reflected in the well-known Stokes law [6].

The modifiers of rheology or thickeners used in many practical applications are polymers, clays, and surfactants [1,2,3,4,5]. However, nanomaterials (nanoparticles and nanocrystals) are emerging as a class of new rheological modifiers and thickeners of liquids. The addition of nanoparticles and/or nanocrystals to liquids can dramatically increase the viscosity of liquids and alter the liquid rheology from Newtonian to shear-thinning non-Newtonian. 

Due to environmental concerns and the shift towards sustainable materials by specialists, bio-based nanomaterials such as nanocrystalline cellulose (NCC), also referred to as cellulose nanocrystals, and starch nanoparticles (SNPs) have emerged as promising nanomaterials that could be used as thickeners and rheology modifiers. These materials are particularly attractive because of their natural abundance, renewability, biodegradability, and compatibility with eco-friendly production processes [7]. They offer potential for functional customization due to their reactive surfaces [8,9]. They could be synthesized via green chemistry routes and have demonstrated efficiency in many applications [10,11,12,13,14,15,16,17,18]. NCC is in the form of rod-shaped nanostructures extracted from the crystalline regions of cellulose microfibrils through “top-down” methods. The most common extraction technique involves acid hydrolysis [19,20,21,22,23,24,25]. Starch nanoparticles (SNPs) are another family of natural nanomaterials with expanding applications. After cellulose, starch is the second most abundant biomass resource, obtained primarily from maize (82%), wheat (8%), potatoes (5%), and cassava (5%) [10,26,27,28,29,30]. Starch nanoparticles are synthesized using both top-down and bottom-up methods such as acid hydrolysis [31,32], nanoprecipitation, and self-assembly [33]. SNPs are widely investigated for food industry applications such as rheological modifiers, emulsion stabilizers, packaging enhancers, and delivery systems for nutraceuticals [31]. Their ability to increase viscosity and reduce sedimentation in food suspensions has been demonstrated, making them effective alternatives to traditional thickeners [34]. They are also used in biomedical and pharmaceutical applications [35]. Thus, NCC and SNPs represent critical advances in green material science. They are used in cosmetics, drug delivery, and sensing due to their surface functionalizability and biocompatibility [21,36].

In this work, NCC, SNPs, and their mixtures are used as thickeners and rheology modifiers of aqueous liquid. The steady rheological behaviors of suspensions of SNPs, NCC, and mixtures of SNPs and NCC are investigated over a broad range of concentrations. To our knowledge, this is the first study exploring the rheology of suspensions of mixtures of SNPs and NCC.

## 2. Materials and Methods

### 2.1. Materials

**Figure 1:**
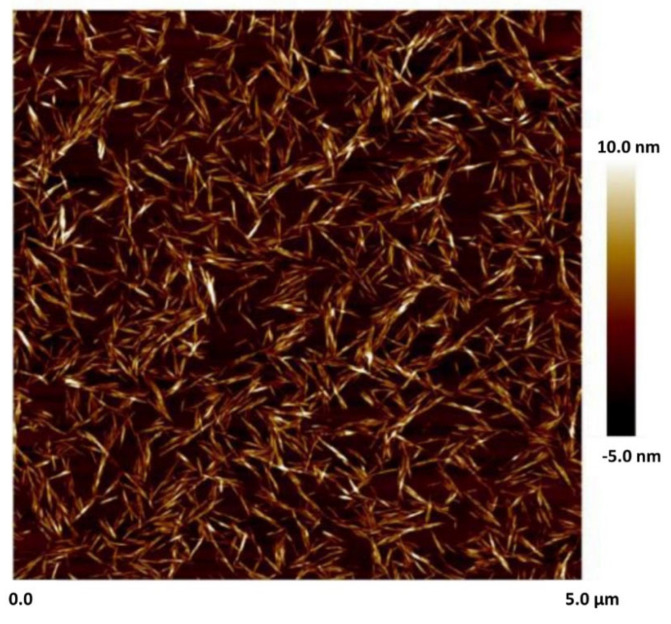
Atomic force microscopy of nanocrystals.

The nanocrystalline cellulose (NCC), also referred to as cellulose nanocrystals, used in this work was available commercially. It was manufactured by CelluForce Inc., Windsor, ON, Canada. The trade name of the supplied NCC was NCC NCV100-NASD90. The method to manufacture NCC involved the sulfuric acid hydrolysis of wood pulp. Figure 1 shows the AFM image of NCC. Clearly, the nanocrystals are rod-shaped particles, based on an AFM image.

The SNPs (starch nanoparticles) used were supplied in powder form by EcoSynthetix Inc., Burlington, ON, Canada. They were produced by modifying native starch using reactive extrusion. They are available commercially and are used extensively in industrial paper and pulp applications.

### 2.2. Preparation of NCC and SNP Suspensions 

The suspensions of nanocrystalline cellulose (NCC) and starch nanoparticles (SNPs) were prepared at room temperature (≅23 °C) in batches of approximately 1 kg. A known amount of NCC or SNP powder was slowly dispersed into a known amount of deionized water while maintaining the mixing of the suspension using a turbine homogenizer (Gifford-Wood, model 1 L, NOV process and flow technologies, Dayton, OH, USA) at a fixed speed. To prevent any bacterial growth in the SNP suspension, a small amount (0.15 wt%) of biocide (Thor Acticide GA) was added. The suspension was homogenized for at least 60 min for the nanocrystals or SNPs to disperse and homogenize fully. Seven differently concentrated suspensions were prepared. For the NCC suspensions, the concentrations were 0.99, 1.97, 2.95, 3.91, 4.86, 5.80 and 6.73 wt%, and for the SNP suspensions, the concentrations were 5, 9.1, 13.05, 16.67, 20, 25 and 30 wt%.

### 2.3. Preparation of Suspensions of SNP and NCC Mixtures

The suspensions of SNP and NCC mixtures were prepared using a two-step procedure. In the first step, SNP suspension was prepared at a fixed SNP concentration. In the second step, a suspension of SNP and NCC mixture was prepared by adding a known amount of NCC powder to an SNP suspension while maintaining the mixing of the suspension. To increase the NCC concentration, the required amount of additional NCC was added to an existing SNP and NCC mixture and the suspension was mixed using a homogenizer at a fixed speed for approximately 60 min. The compositions of the suspensions of SNP and NCC mixtures investigated in this study are given in Table 1.

### 2.4. Measurements

For the measurement of the steady rheology of suspensions, two viscometers, Fann and Haake viscometers, were used. Fann and Haake viscometers are co-axial cylinder type viscometers. The dimensions of the co-axial cylinders and gap widths utilized are given in Table 2. Note that the inner cylinder is kept stationary and the outer cylinder rotates in a Fann viscometer, whereas the inner cylinder rotates, and the outer cylinder is held stationary in a Haake viscometer. There are 12 speeds in the Fann viscometer ranging from 0.9 to 600 rpm. In the Haake viscometer, there are 30 speeds ranging from 0.01 to 512 rpm. Standards of known viscosities were used to calibrate the viscometers. All rheological measurements were carried out at room temperature.

Dynamic Light Scattering (DLS) was employed to determine the size distributions of starch nanoparticles (SNPs) and nanocrystalline cellulose (NCC). The measurements were conducted using a Zetasizer Nano ZS90 instrument manufactured by Malvern Instruments Ltd, Worcester, UK. Specifically, the Zetasizer 6.20 software was utilized for both data acquisition and analysis. The SNP and NCC samples, comprising dilute suspensions of SNPs and NCC in water, were tested in ZEN0112 low-volume disposable cuvettes, and analyzed at a standard temperature of 25 °C. Prior to analysis, a 120 s period of equilibration was observed to ensure optimal sample stability. 

## 3. Results and Discussion

### 3.1. Particle Size Analysis of NCC and SNPs

**Figure 2:**
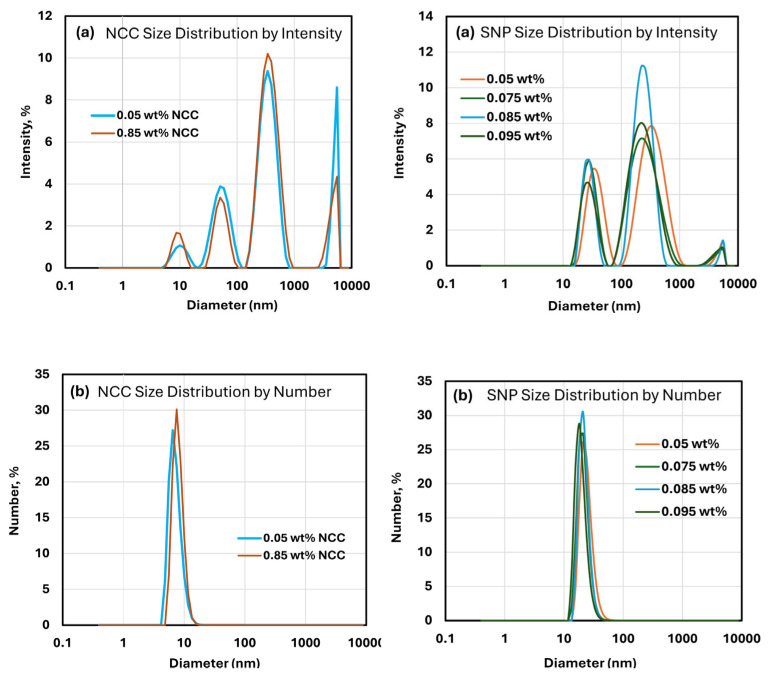
Dynamic Light Scattering (DLS) of suspensions of cellulose nanocrystals and starch nanoparticles at different concentrations. (**a**) Intensity distribution of nanocrystals and nanoparticles; (**b**) number distribution of nanocrystals and nanoparticles.

Figure 2 shows the DLS data for NCC and SNP suspensions at different concentrations. The intensity distributions shown in Figure 2a exhibit several peaks indicating aggregation of nanocrystals and nanoparticles. The smallest peaks correspond to primary unaggregated nanocrystals and nanoparticles. The number distributions shown in Figure 2b exhibit only a single peak corresponding to primary unaggregated nanocrystals and nanoparticles. The average hydrodynamic diameter of primary unaggregated NCC based on Figure 2b is approximately 7.6 nm, whereas the average hydrodynamic diameter of primary unaggregated SNPs based on Figure 2b is approximately 21.7 nm.

Note that the number distributions of DLS emphasize the species with the highest number of particles, which are the smaller primary particles. The intensity distributions of DLS emphasize the species with the largest scattering intensity, which are the larger aggregated particles. For rod-shaped NCC, the DLS measurement gives an equivalent hydrodynamic diameter, not the actual dimensions of NCC. Based on an AFM image (see Figure 1), the mean length and mean width of NCC are 76 nm and 3.4 nm, respectively.

### 3.2. Rheology of Suspensions of NCC

**Figure 3:**
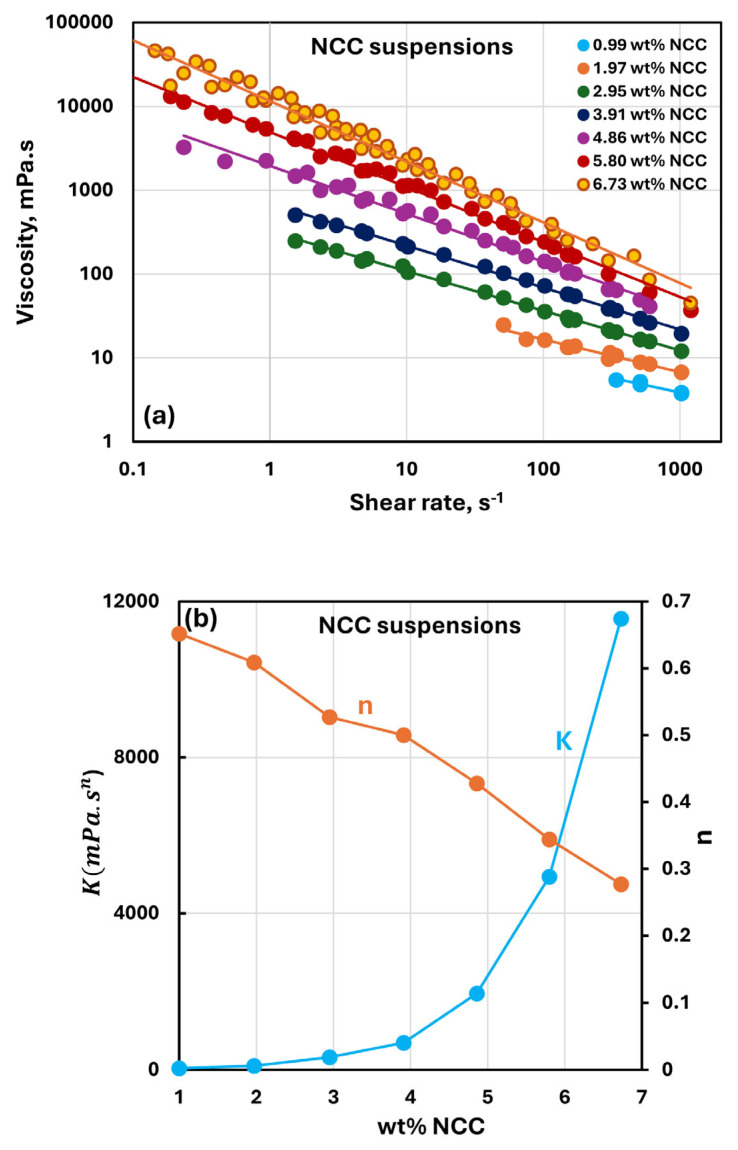
Rheology of suspensions of nanocrystalline cellulose (NCC). (**a**) Viscosity versus shear rate plots of NCC suspensions; (**b**) power-law constants of NCC suspensions.

Figure 3 shows the rheological behavior of differently concentrated suspensions of nanocrystalline cellulose (NCC). The concentration of NCC varies from 0.99 to 6.73 wt%. Viscosity decreases with an increase in shear rate, indicating that all NCC suspensions are shear-thinning in nature. With an increase in NCC concentration, the viscosity increases. All the viscosity versus shear rate data could be described by a power-law model [37]:

(1) $$\tau =K{\dot{\gamma }}^{n}$$

(2) $$\eta =\tau /\dot{\gamma }=K{\dot{\gamma }}^{n-1}$$

where τ is the shear stress, $\dot{\gamma }$ is the shear rate, K is the consistency index, n is the flow behavior index, and η is the viscosity. K and n together are referred to as power-law constants. K is a measure of the consistency of fluid and n is a measure of the flow behavior (Newtonian versus non-Newtonian) of fluid. For Newtonian fluids, n=1. For shear-thinning fluids, n<1.

According to the power-law model, the plots of viscosity versus shear rate are linear on a log–log scale, as shown in Figure 3a. Power-law constants K and n are shown in Figure 3b as functions of NCC concentration. With an increase in NCC concentration, the consistency index K rises sharply and the flow behavior index n decreases. Thus, NCC suspensions become more viscous and shear-thinning with an increase in NCC concentration.

### 3.3. Rheology of Suspensions of SNPs

**Figure 4:**
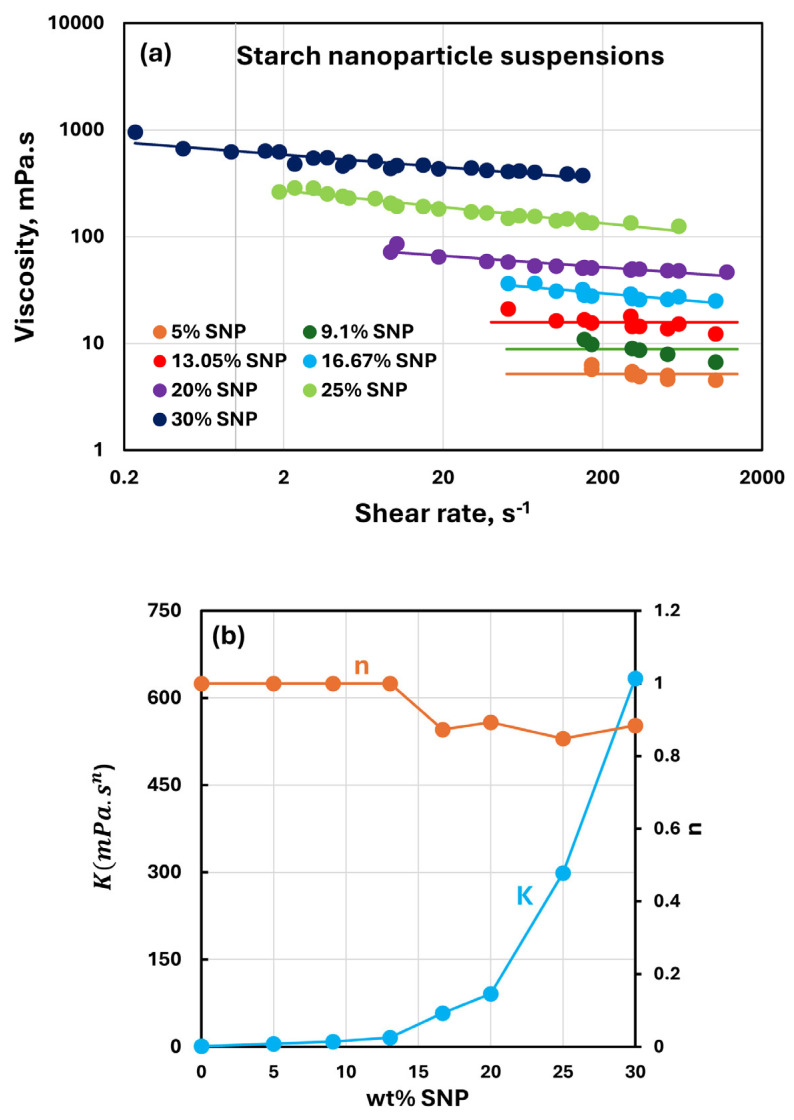
Rheology of suspensions of starch nanoparticles (SNPs). (**a**) Viscosity versus shear rate plots of SNP suspensions; (**b**) power-law constants of SNP suspensions.

Figure 4 shows the rheological behavior of differently concentrated suspensions of starch nanoparticles (SNPs). The concentration of SNPs varies from 5 to 30 wt%. Compared with NCC suspensions, SNP suspensions are only slightly shear-thinning at high SNP concentrations (>13 wt%). With an increase in SNP concentration, the viscosity increases, as reflected in the consistency index K. The flow behavior index n is unity at SNP concentrations less than 13 wt%, indicating Newtonian behavior. At SNP concentrations higher than 13 wt%, the flow behavior index is only slightly less than unity, indicating modest shear-thinning behavior.

### 3.4. Rheology of Suspensions of Mixtures of NCC and SNPs

**Figure 5:**
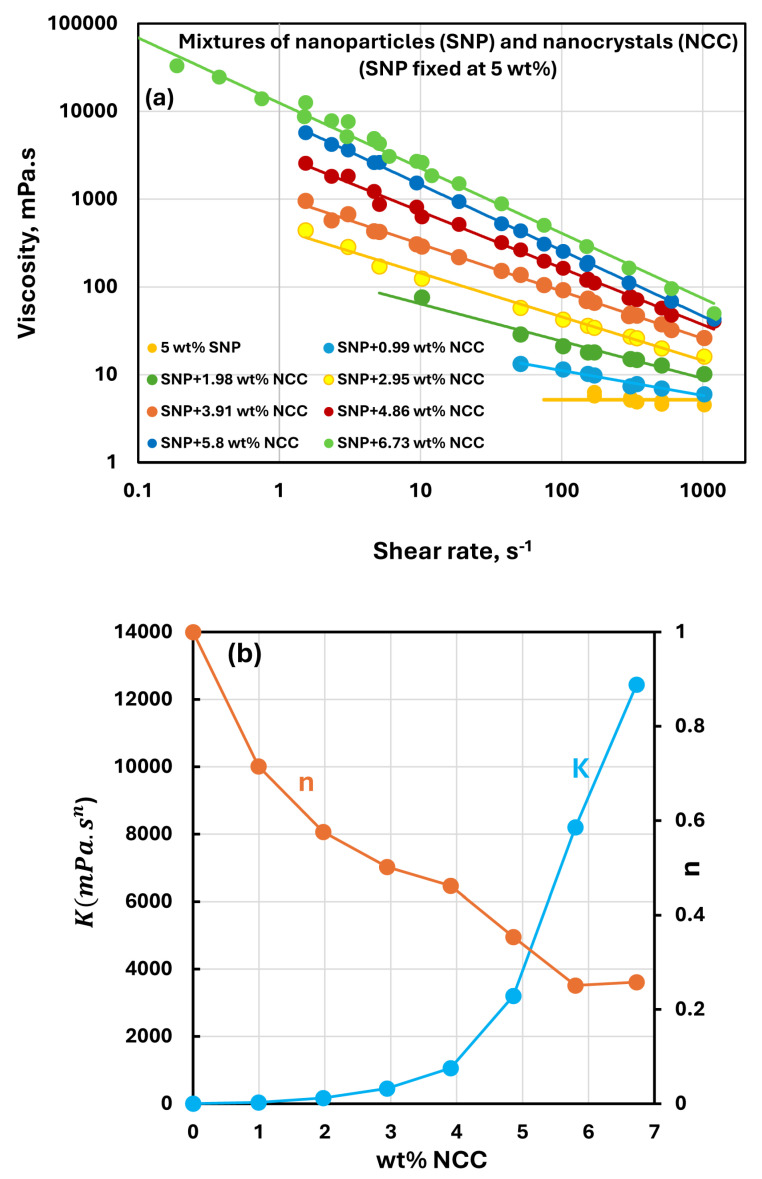
Rheological behavior of suspensions of mixtures of NCC and SNPs at a fixed SNP concentration of 5 wt%. (**a**) Viscosity versus shear rate plots of suspensions of mixtures of NCC and SNPs; (**b**) power-law constants of suspensions of mixtures of NCC and SNPs.

**Figure 6:**
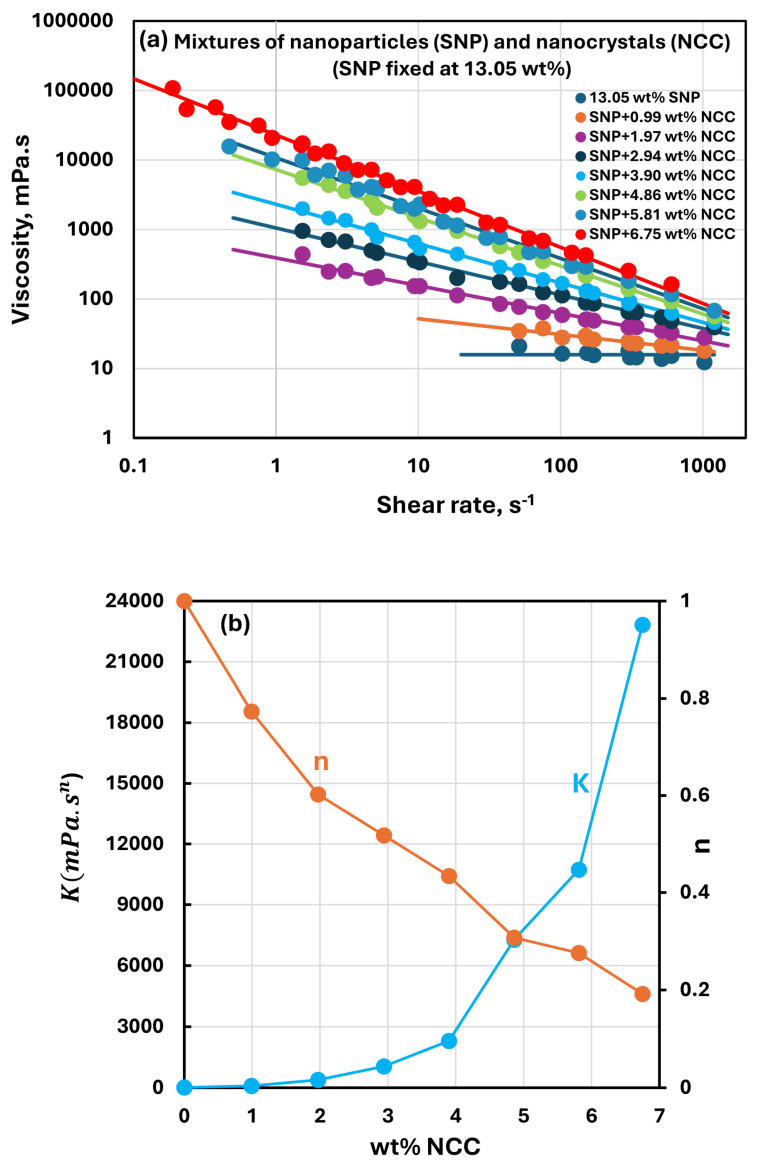
Rheological behavior of suspensions of mixtures of NCC and SNPs at a fixed SNP concentration of 13.05 wt%. (**a**) Viscosity versus shear rate plots of suspensions of mixtures of NCC and SNPs; (**b**) power-law constants of suspensions of mixtures of NCC and SNPs.

**Figure 7:**
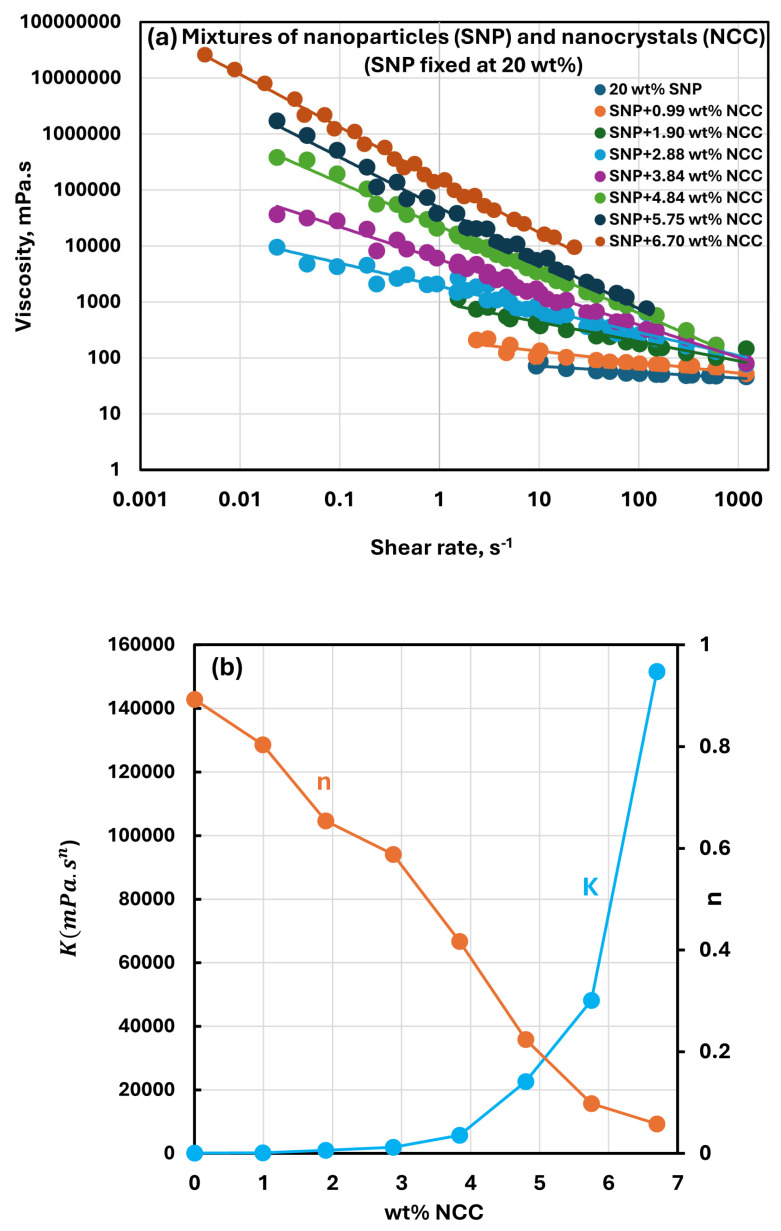
Rheological behavior of suspensions of mixtures of NCC and SNPs at a fixed SNP concentration of 20 wt%. (**a**) Viscosity versus shear rate plots of suspensions of mixtures of NCC and SNPs; (**b**) power-law constants of suspensions of mixtures of NCC and SNPs.

**Figure 8:**
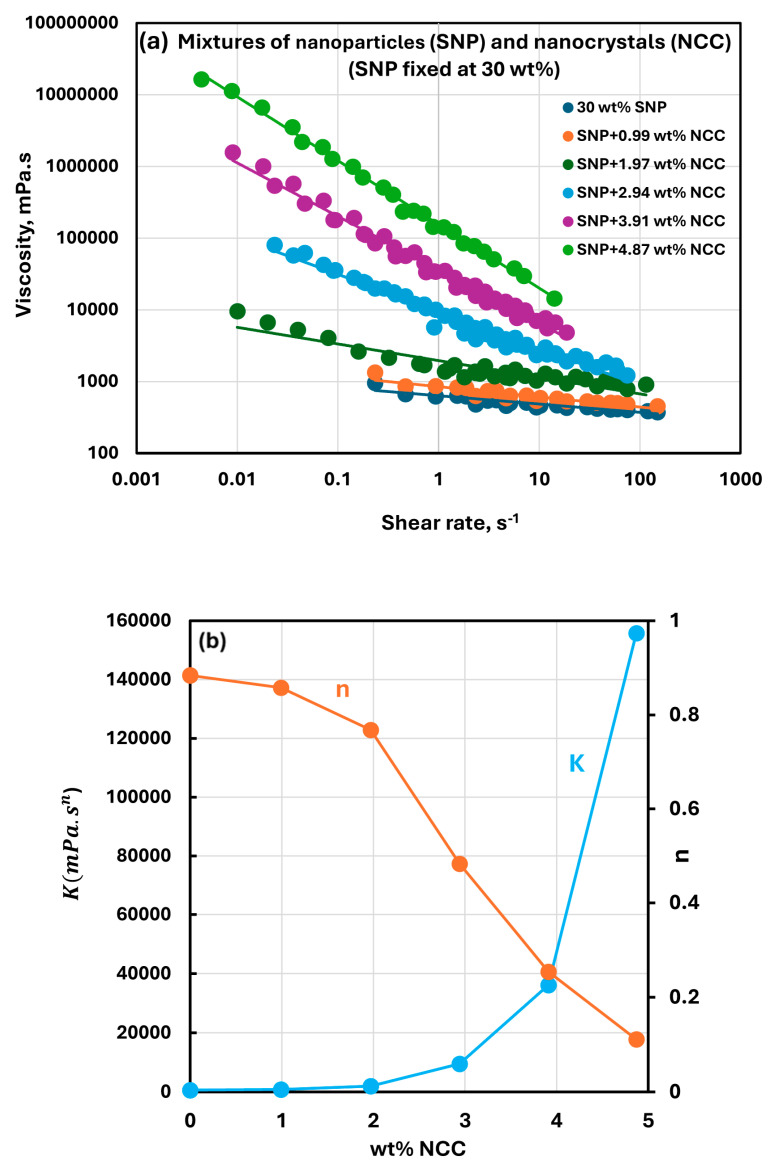
Rheological behavior of suspensions of mixtures of NCC and SNPs at a fixed SNP concentration of 30 wt%. (**a**) Viscosity versus shear rate plots of suspensions of mixtures of NCC and SNPs; (**b**) power-law constants of suspensions of mixtures of NCC and SNPs.

Figure 5, Figure 6, Figure 7 and Figure 8 show the rheological behavior of sample suspensions of mixtures of NCC and SNPs. In part (a) of each figure, the viscosity versus shear rate data is plotted at different NCC concentrations at a fixed SNP concentration. As the viscosity versus shear plots are linear on a log–log scale, the suspensions of mixtures of NCC and SNPs follow the power-law model (Equations (1) and (2)). The power-law constants, consistency index K, and flow behavior index n are plotted in part (b) of each figure as functions of NCC concentration at a fixed SNP concentration. Figure 5, Figure 6, Figure 7 and Figure 8 reveal the following characteristics of the rheological behaviors of suspensions of mixtures of NCC and SNPs:

- The suspensions of mixtures of NCC and SNPs are Newtonian at low concentrations of NCC and SNPs in the mixture suspension. The mixture suspensions become shear-thinning pseudoplastic, that is, the viscosity decreases with an increase in shear rate, at high concentrations of NCC and SNPs in the mixture suspension.
- At a fixed concentration of SNPs, the mixture suspensions become more viscous and shear-thinning with an increase in NCC concentration. In other words, the viscosity versus shear rate plot shifts upwards and becomes steeper with an increase in NCC concentration. The consistency index K increases whereas the flow behavior index n decreases with an increase in the NCC concentration of the mixture suspension.
- The consistency index K of suspensions of mixtures of NCC and SNPs is strongly dependent on both NCC and SNP concentrations of the mixtures. For example, the consistency index increases substantially with an increase in SNP concentration at any fixed concentration of NCC.

**Figure 9:**
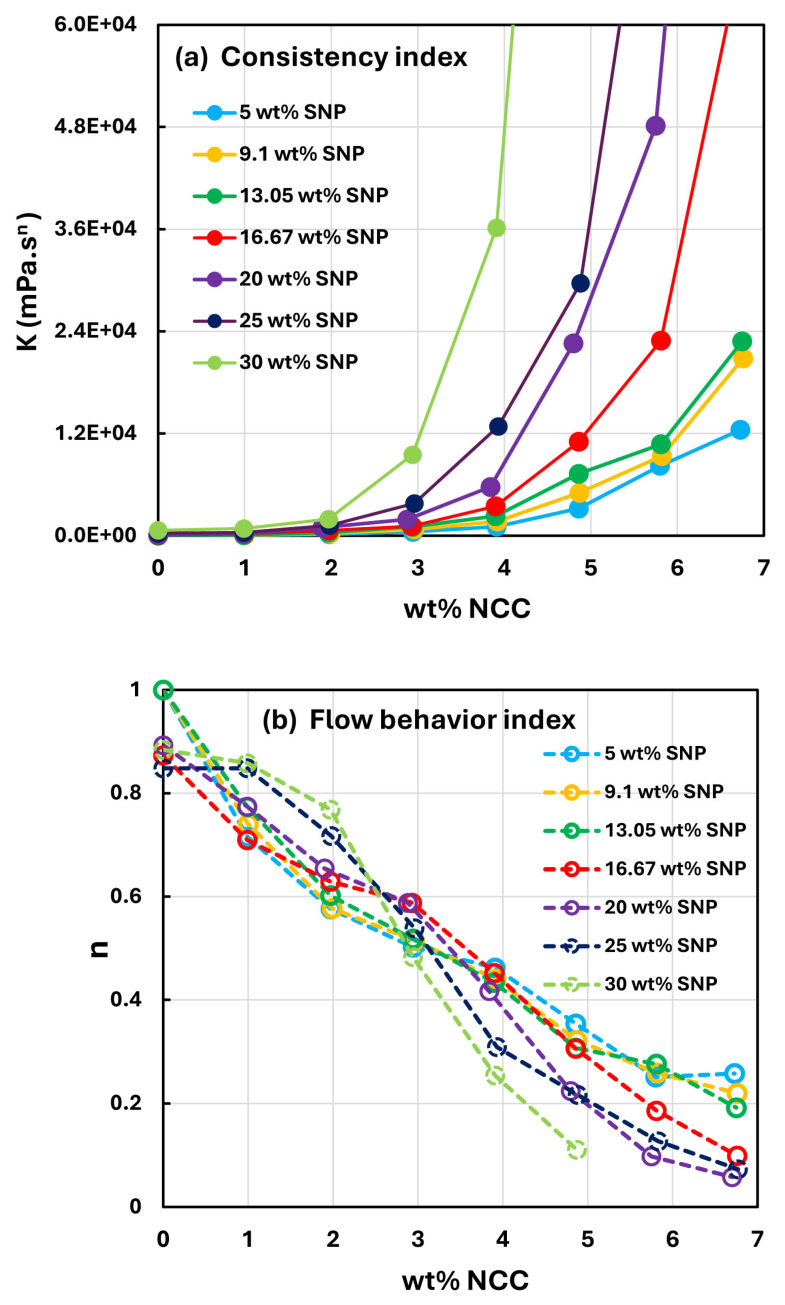
Comparison of the rheological characteristics of suspensions of NCC and SNP mixtures. (**a**) Comparison of consistency index; (**b**) comparison of flow behavior index.

The power-law parameters, consistency index K and flow behavior index n for suspension mixtures of NCC and SNP are compared in Figure 9. Figure 9a compares the consistency index in that the consistency index is plotted as a function of NCC concentration for different values of SNP concentration. At any given SNP concentration, the consistency index rises sharply with an increase in NCC concentration, especially at high NCC concentrations. With an increase in SNP concentration, the consistency index versus NCC concentration plot shifts upwards, indicating an increase in consistency index with an increase in SNP concentration. Figure 9b compares the flow behavior index of different suspension mixtures of NCC and SNP. With an increase in NCC concentration, the flow behavior index decreases, indicating that the degree of shear-thinning of suspensions of NCC-SNP mixtures increases with an increase in the NCC content of the mixture. No clear trend is observed in the variation of flow behavior index with an increase in SNP concentration. Thus, the non-Newtonian character of suspensions of mixtures of NCC and SNPs is controlled by the NCC content of the mixture.

**Figure 10:**
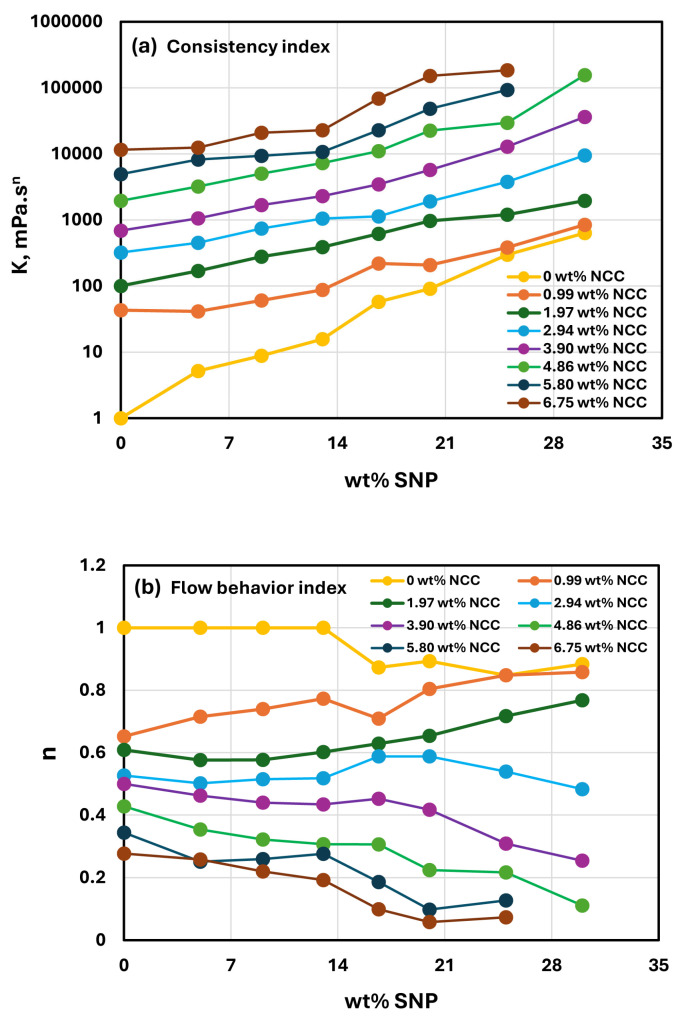
(**a**) Comparison of consistency index versus SNP concentration for different values of NCC concentration; (**b**) comparison of flow behavior index versus SNP concentration for different values of NCC concentration.

Figure 10 compares the power-law constants of suspensions of mixtures of NCC and SNPs as functions of SNP concentration while keeping the NCC concentration fixed. As indicated in Figure 10a, the consistency index increases almost linearly (on a semi-log plot) with an increase in SNP concentration at any given NCC concentration. With an increase in NCC concentration, the consistency index versus SNP concentration plot shifts upwards, indicating an increase in the consistency index with an increase in NCC concentration. Figure 10b shows the plots of flow behavior index as a function of SNP concentration for different values of NCC concentration. The flow behavior index does not show a clear trend with SNP concentration variation at low values of NCC concentration. At high concentrations of NCC (≥3.9 wt% NCC), the flow behavior index decreases with an increase in SNP concentration. However, the flow behavior index versus SNP concentration plot shifts downwards to lower n values with an increase in NCC concentration. Thus, the suspensions of mixtures of NCC and SNP become more shear-thinning with an increase in NCC concentration.

**Figure 11:**
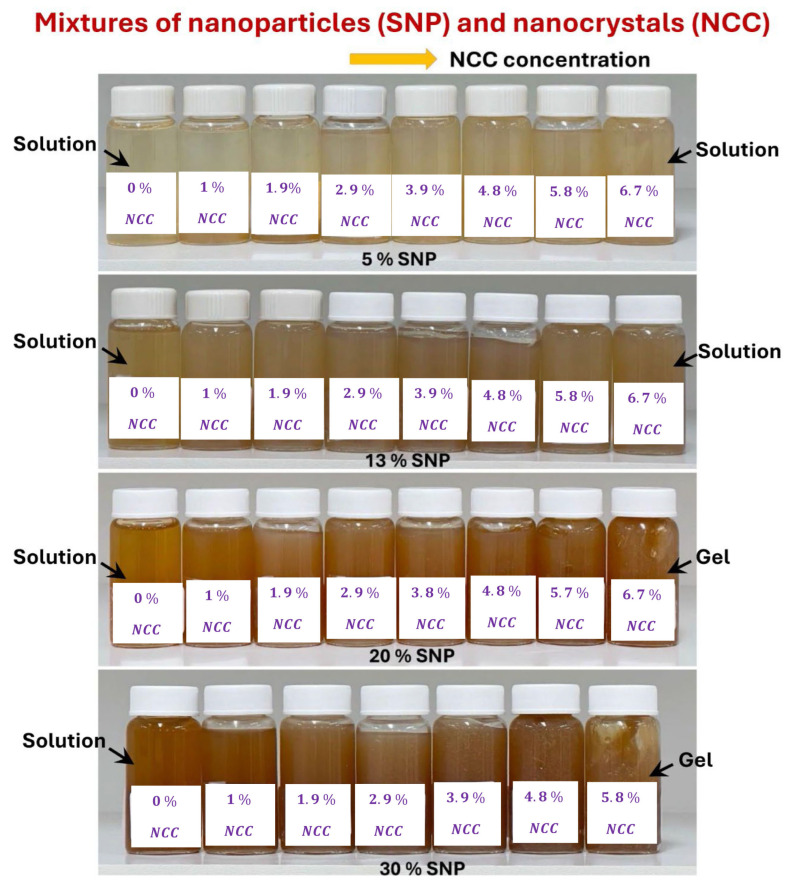
Samples of suspensions of mixtures of SNPs and NCC.

Figure 11 shows pictures of samples of suspensions of mixtures of NCC and SNP. At low concentrations of NCC and SNP, the consistency of the suspension mixtures is a homogeneous solution. However, at high concentrations of NCC and SNP, the suspension mixtures become more like gels.

## 4. Conclusions

The viscous behavior of suspensions of mixtures of cellulose nanocrystals (NCC) and starch nanoparticles (SNPs) was investigated experimentally. Based on experimental work, the following conclusions can be drawn:

- The NCC suspensions are shear-thinning and follow a power-law model over the NCC concentration range of 0.99 to 6.73 wt%. The consistency and the degree of shear-thinning of suspensions both increase with an increase in NCC concentration.
- The SNP suspensions are Newtonian up to an SNP concentration of about 13 wt%. At higher SNP concentrations, suspensions become modestly shear-thinning. However, the consistency index continues to increase substantially with an increase in SNP concentration.
- The suspensions of mixtures of NCC and SNPs are non-Newtonian shear-thinning over the entire range of concentrations investigated. The degree of shear-thinning in suspensions of mixtures of NCC and SNPs is strongly dependent on the NCC concentration and less severely dependent on the SNP concentration. The consistency of the suspension mixture increases substantially with increases in NCC and SNP concentrations. However, NCC concentration has a stronger effect on consistency as compared with SNP concentration.
- Our future work in this area will explore dynamic (oscillatory) shear properties of suspensions of NCC, SNPs, and their blends.

## Figures and Tables

**Table 1:** Compositions of suspensions of SNP and NCC mixtures investigated.

Starch Nanoparticle (SNP) Concentration in SNP–Water Suspension (wt%)	Nanocrystalline Cellulose (NCC) Concentration in NCC–SNP–Water Suspension (wt%)
5.0	Seven concentrations: 0.99, 1.98, 2.95, 3.91, 4.86, 5.80, 6.73
9.1	Seven concentrations: 1.0, 1.99, 2.96, 3.92, 4.87, 5.82, 6.76
13.05	Seven concentrations: 0.99, 1.97, 2.94, 3.90, 4.86, 5.81, 6.75
16.67	Seven concentrations: 0.99, 1.97, 2.93, 3.90, 4.86, 5.81, 6.76
20.0	Seven concentrations: 0.99, 1.90, 2.88, 3.84, 4.84, 5.75, 6.70
25.0	Seven concentrations: 0.99, 1.98, 2.96, 3.93, 4.88, 5.83, 6.77
30.0	Five concentrations: 0.99, 1.97, 2.94, 3.91, 4.87

**Table 2:** Dimensions of co-axial cylinders and gap widths used in viscometers.

Device	$\mathrm{Inner}\;\mathrm{Cylinder}\;\mathrm{Radius},\;R_{i}$ (cm)	$\mathrm{Outer}\;\mathrm{Cylinder}\;\mathrm{Radius},\;R_{o}\;(cm)$	Length of Inner Cylinder (cm)	Gap-Width (cm)
Fann 35A/SR-12	1.72	1.84	3.8	0.12
Haake Roto-visco RV 12 with MV I	2.00	2.1	6.0	0.10
Haake Roto-visco RV 12 with MV II	1.84	2.1	6.0	0.26
Haake Roto-visco RV 12 with MV III	1.52	2.1	6.0	0.58

## Data Availability

The raw data supporting the conclusions of this article will be made available by the authors on request.
